# Empirical Analysis and Suggestions on the Selection of City Leading Industries based on SSM Algorithm

**DOI:** 10.1155/2022/9337569

**Published:** 2022-05-06

**Authors:** Junhang Lin, Yong Liu, Hua Tang, Huipeng Zeng, Shiyuan Li

**Affiliations:** ^1^Faculty of Architecture and Urban-Rural Planning, Zhuhai College of Science and Technology, Zhuhai 519041, Guangdong, China; ^2^Faculty of Innovation and Design, City University of Macau, Macau 999078, China; ^3^Faculty of Xunxing Architecture, Quanzhou College of Technology, Quanzhou 362200, Fujian, China; ^4^School of Architecture and Civil Engineering, Huizhou University, Huizhou 516007, Guangdong, China; ^5^IUAV University of Venice, 30135 Venice, Italy

## Abstract

City leading industries are the pillars of urban economic development and are constantly changing as urban economic development enters different stages. The weight setting of many factors in the existing leading industry selection methods and means is mainly set by humans, which is highly subjective and lacks dynamics, integrity, and quantification, and the accuracy of prediction results is not high. Therefore, starting from respecting objective data, the SSM selection method with both dynamic and quantifiable properties is introduced. Based on the SSM mathematical model and principles, 35 manufacturing industries in Guangzhou in 2015 and 2020 are selected as initial variables and stage variables, respectively, taking 35 corresponding industrial sectors in the province as reference variables at the same time point and using the SSM algorithm as an analytical tool to conduct an empirical analysis of the share deviation component, structural deviation component, and competitiveness deviation component of the 35 manufacturing industry sectors in Guangzhou. After drawing the Shift-share analysis chart, it was found that there are 12 industrial sectors most likely to become the city leading industries in Guangzhou, and 4 suggestions for the development planning of city leading industries were put forward; they are, respectively, ➀ accelerate traditional industries technological upgrading, ➁ focus on optimizing automobile manufacturing industry, ➂ promote leading industries independent innovation, and ➃ create leading industry sharing platform.

## 1. Introduction

The concept and theory of leading industry, also translated as leading sector, was first proposed by the American economic historian and development economist W. W. Rostow [1], and he defines an industry with “high growth rate while driving the growth of other industrial sectors” as a leading industry [2]. City leading industries are the industries or industrial groups which not only have broad market prospects and strong potential for technological progress in a certain stage of a urban economic development but also can maintain high-speed growth and sustainable and stable development in the future and at the same time can promote the overall rise and drive of the urban economy [3]. They not only have significant industrial scale and strong correlation effect but also have strong innovation ability, good development potential, and continuous sectoral growth advantages. Their advantageous sectors play an important role in optimizing urban industrial structure and stimulating urban economic development [4]. The selection of city leading industries refers to determining the sequence of industrial development according to a certain stage of urban economic development, so as to realize the rationalization and advancedization of the industrial structure [5]. Therefore, how to accurately select and determine the leading industry has a great significance to the healthy development of the urban economy. Then, it is very important to adopt a comprehensive dynamic and efficient selection method, so the SSM selection method is introduced.

## 2. Theory and Research Method of Urban Leading Industry Selection

### 2.1. Overview of Leading Industry Theories and Selection Datums

The origin of the leading industry theory can be traced back to the classical economic era. In 1817, the British political economist D. Ricardo put forward the “Theory of Comparative Advantage” in his book “*Principles of Political Economy and Taxation*” [6]. Subsequently, scholars from various countries put forward theories on the choice of leading industries based on the historical background and human environment of their own countries. American economist Albert Hirschman is the most representative theoretical scholar in industrial theory research [7]. In 1958, Albert put forward the “Industry Correlation Theory” of the selection of leading industries in his book “*Economic Development Strategy*” [8]. In 1960, the American economic historian and development economist W. W. Rostow put forward the “Industrial Diffusion Effect Theory” and the “Phase Theory of Economic Growth” in his book “Phases of Economic Growth” [9]. In 1990, Michael E. Porter, a professor of strategic management scientist at Harvard University in the United States, put forward the “diamond theory” of the selection of leading industries in his book “*National Competitive Advantage*” [10].

The development of the leading industry theory needs to answer the question of how to choose the leading industry, so many economists put forward various datums for the selection of the leading industry. In 1933, Swedish economists E. E. Heckscher and B. Ohlin put forward the “resource endowment datum” [11]. In 1957, Japanese industrial economist Shinohara Sandaihei put forward the “demand income elasticity datum” and “productivity rate datum,” namely, the famous “Shinohara II datum” [12]. In 1958, the American development economist Albert Hirschman put forward the “Industrial Correlation Effect Datum” [13]. In 1960, the American economic historian and development economist W. W. Rostow put forward the “growth rate datum” [14]. In 1971, the Japan Industrial Structure Council put forward the “Environmental Criteria” and “Enriched Labor Content Criteria” [15]. In 1986, American economists HB Chenery, S. Robinson, and M. Syrquin put forward the “Chenery-Robinson-Syrquin datum” [16]. Since then, Chinese scholars have put forward many different selection criteria based on China's special national conditions and the characteristics of regional economy. In 1990, Zhou Shulian, Pei Shuping, and Chen Shuxun put forward the “marginal savings rate datum” [17]. In 1991, Zhou Zhenhua put forward the three datums of “growth stamina, elasticity of shortage substitution, and bottleneck effect” [18]. In 1996, Liu Zaixing put forward the “two-way datum of comprehensive method” [19]. In 1998, He Jingming and Lu Xu put forward the four datums of “market demand, industrial relevance, resource efficiency, and economic benefit comparison” [20]. In 2002, Guan Aiping and Wang Yu put forward six datums of “sustainable development, market, efficiency, technological progress, industrial relevance, and competitive advantage” [21]. In 2005, Xie Zhihong put forward the five datums of “market potential, regional comparative advantage, industrial relevance, resource and environment level, and innovation ability” [22]. In 2009, Hu Jianji and Zhang Jin put forward two datums of “industrial location competitiveness and development potential” [23]. In 2014, Liu Jing put forward three datums of “market structure, production factors, and energy consumption” [24].

To sum up, the research on the selection of leading industries by Western academic experts focuses on the contribution of industries in pure economic aspects, and the proposed datum for selection of leading industries is basically the datum in pure economic aspects. With the development of the economy and the changes of the social and economic environment, it will no longer meet the new requirements of the new industrialization road for the selection of leading industries. However, Chinese scholars' research on the selection of leading industries mainly focuses on framework research and is highly subjective in index selection and comprehensive evaluation, and research on the datums, principles, and index systems for the selection of leading industries is not perfect enough, and especially in the artificial and subjective setting of the weights of various factors in the quantification method it leads to low decision-making accuracy [25].

### 2.2. Research Methods and Mathematical Models

In view of the fact that most of the existing leading industry selection datum methods only reflect the static characteristics of industrial sectors from different aspects and their selection indicators are more arbitrary and independent, they cannot form a complete system, and the calculation methods are too complicated and cannot well reflect the dynamic characteristics of the industrial sector [26]. Therefore, a dynamic method, “Shift-share Method (SSM),” which can simultaneously reflect the important characteristics of industrial sectors' development prospects, structural foundation, and competitiveness, is introduced. The Shift-share Method was successively proposed by American scholars Dunn, Perloff, Lampard, and Muth in the 1960s and was summed up by Dunn's collection of various directors about the selection method of regional leading industries in the 1980s. SSM regards the development and changes of the regional economy as a dynamic process and takes the economic development of the large region (province) or the whole country where the region (city) is located as the reference object and decomposes the change of the region's own economic aggregate in a certain period into three parts: the share deviation component, the structural deviation component, and the competitiveness deviation component, based on which to analyze the reasons for regional economic development or decline, evaluate the advantages and disadvantages of the regional industrial structure and the competitiveness of each industry, finding out the industry with relative competitive advantage in the region as the leading industry, and then determine the reasonable direction of the future economic development of the region and formulate the policy of industrial structure adjustment.

The mathematical model and principle of SSM [27] are as follows.

Suppose that, after the time period [0, *t*] in region *i*, the economic aggregate and structure have changed. Let the total economic size of region *i* in the initial year be *b*_*i*,0_, The total size of the economy by the end of the year is *b*_*i*,*t*_. Divide the regional economy into *n* industrial sectors, take *b*_*ij*,0_ and *b*_*ij*,*t*_ (*j* = 1, 2, 3,…, *n*) to, respectively, represent the economic scale of the *j*th industrial sector in region *i* in the initial year and the end year, and take *B*_0_ and *B*_*t*_ to, respectively, represent the total economic scale of the large region or the whole country in the initial year and the final year where region *i* is located, and then take *B*_*j*,0_ and *B*_*j*,*t*_ to, respectively, represent the economic scale of the *j*th industrial sector in the initial year and the end year of the large region or the whole country where region *i* is located. Then we have the following.

The rate of change of the *j*th industrial sector in region *i* in the time period [0, *t*] is

(1) $$\begin{array}{c} r_{ij}=\frac{b_{ij,t}-b_{ij,0}}{b_{ij,0}},\;j=1,2,3,\ldots ,n. \end{array}$$

The change rate of the large region or the whole country of the *j*th industrial sector in the time period [0, *t*] where region *i* is located is

(2) $$\begin{array}{c} R_{j}=\frac{B_{j,t}-B_{j,0}}{B_{j,0}},\;j=1,2,3,\ldots ,n. \end{array}$$

Standardizing the economic scale of each industrial sector in region *i* by the share of region *i* in each industrial sector in the large region or the country, we can get

(3) $$\begin{array}{c} b_{ij}^{\prime }=\frac{b_{ij,0}\cdot B_{j,0}}{B_{j,0}},\;j=1,2,3,\ldots ,n. \end{array}$$

Therefore, the growth amount *G*_*ij*_ of the *j*th industrial sector in region *i* in the time period [0, *t*] can be decomposed into three components: the share deviation component *N*_*ij*_, the structural deviation component *P*_*ij*_, and the competitiveness deviation component *D*_*ij*_, respectively:

(4) $$\begin{array}{c} G_{ij}=N_{ij}+P_{ij}+D_{ij}, \end{array}$$

(5) $$\begin{array}{c} N_{ij}=b_{ij}^{\prime }\cdot R_{j}, \end{array}$$

(6) $$\begin{array}{c} P_{ij}=\left(b_{ij,0}-b_{ij}^{\prime }\right)\cdot R_{j}, \end{array}$$

(7) $$\begin{array}{c} D_{ij}=\left(r_{ij}-R_{j}\right)\cdot b_{ij,0}, \end{array}$$

(8) $$\begin{array}{c} PD_{ij}=P_{ij}+D_{ij}. \end{array}$$

Therefore, the economic increment *G*_*i*_ of region *i* is

(9) $$\begin{array}{c} G_{i}=N_{i}+P_{i}+D_{i}, \end{array}$$

(10) $$\begin{array}{c} N_{i}=\sum_{j=1}^{n}b_{ij}^{\prime }\cdot R_{j}, \end{array}$$

(11) $$\begin{array}{c} P_{i}=\sum_{j=1}^{n}\left(b_{ij,0}-b_{ij}^{\prime }\right)\cdot R_{j}, \end{array}$$

(12) $$\begin{array}{c} D_{i}=\sum_{j=1}^{n}\left(r_{ij}-R_{j}\right)\cdot b_{ij,0}. \end{array}$$

In order to evaluate the overall industrial structure characteristics of region *i*, let

(13) $$\begin{array}{c} K_{j,0}=\frac{b_{ij,0}}{B_{j,0}}, \end{array}$$

(14) $$\begin{array}{c} K_{j,t}=\frac{b_{ij,t}}{B_{j,t}}, \end{array}$$

where *K*_*j*,0_ and *K*_*j*,*t*_, respectively, are the proportions of the *j*th industrial sector in region *i* in the initial year and the end year in the corresponding sector in the same period of the large region or the whole country, introducing the regional relative growth rate index *L*, the regional structural effect index *W*, and the regional competition effect index *u*. Then there are

(15) $$\begin{array}{c} L=\frac{\sum_{j=1}^{n}K_{j,t}\cdot B_{j,t}}{\sum_{j=1}^{n}K_{j,0}\cdot B_{j,0}}:\frac{\sum_{j=1}^{n}B_{j,t}}{\sum_{j=1}^{n}B_{j,0}}=\left[\frac{\sum_{j=1}^{n}K_{j,0}\cdot B_{j,t}}{\sum_{j=1}^{n}K_{j,0}\cdot B_{j,0}}:\frac{\sum_{j=1}^{n}B_{j,t}}{\sum_{j=1}^{n}B_{j,0}}\right]\cdot \left[\frac{\sum_{j=1}^{n}K_{j,t}\cdot B_{j,t}}{\sum_{j=1}^{n}K_{j,0}\cdot B_{j,t}}\right]=W\cdot u. \end{array}$$

That shows

(16) $$\begin{array}{c} W=\left[\frac{\sum_{j=1}^{n}K_{j,0}\cdot B_{j,t}}{\sum_{j=1}^{n}K_{j,0}\cdot B_{j,0}}:\frac{\sum_{j=1}^{n}B_{j,t}}{\sum_{j=1}^{n}B_{j,0}}\right], \end{array}$$

(17) $$\begin{array}{c} u=\left[\frac{\sum_{j=1}^{n}K_{j,t}\cdot B_{j,t}}{\sum_{j=1}^{n}K_{j,0}\cdot B_{j,t}}\right]. \end{array}$$

### 2.3. SSM Algorithm Analysis and Three Selection Datums

Deduction of the SSM mathematical model shows the following: If *G*_*i*_ is larger and *L* > 1, the economic growth of region *i* is faster than the large region or the whole country where it is located; otherwise, it is slower than the large region or the whole country. If *P*_*i*_ is large and *W* > 1, region *i* contains a relatively large proportion of the sunrise industry sector, the overall economic structure of region *i* is relatively good, and the structure contributes greatly to economic growth; otherwise, region *i* goes through economic recession, the proportion of sunset sector is relatively large, and the economic structure needs to be adjusted. If *D*_*i*_ is larger and *u* > 1, then there are more industrial sectors in region *i*, which are developing rapidly, their status is rising, and they have strong competitiveness; otherwise, there are more industrial sectors in region *i*, whose development is slow, their status is declining, and their competitiveness is weak. Therefore, in the selection process of regional leading industries, in order to more fully reflect the dynamic characteristics of various industrial sectors, the SSM can be decomposed into three selection datums.

#### 2.3.1. Share Deviation Selection Datum

The share deviation selection datum is derived from the share deviation component *N*_*ij*_, which represents the economic development trend of the corresponding industrial sector in region *i*. A positive value indicates that the industrial sector has good economic development prospects in the region, and the larger the positive value, the better the development prospects.

#### 2.3.2. Structural Deviations Selection Datum

The structural deviations selection datum is derived from the structural deviation component *P*_*ij*_, which represents the industrial structure basis of the corresponding industrial sector in region *i*. A positive value indicates that the industrial sector has a good industrial structure foundation in the region, and the larger the positive value, the better the structural foundation.

#### 2.3.3. Competitiveness Deviation Selection Datum

The competitiveness deviation selection datum is derived from the competitiveness deviation component *D*_*ij*_, which represents the relative competitiveness of the corresponding industrial sector in region *i*. A positive value indicates that the industrial sector grows faster than the corresponding industrial sector in the large region or the whole country where it is located and has strong competitiveness, and the larger the positive value, the stronger the competitiveness.

## 3. Study Samples and Empirical Analysis

### 3.1. Selecting Research Samples

On January 20, 2022, the Guangdong Bureau of Statistics released data on the economic operation of Guangdong in 2021. According to the unified accounting results of the regional GDP, the GDP of Guangdong in 2021 was 12,436.967 billion yuan, a year-on-year increase of 8.0% and an average growth rate of two years of 5.1% [28], becoming the first province in China with a GDP exceeding 12 trillion yuan, ranking first in China for 33 consecutive years in terms of GDP. However, Guangzhou, the capital city of Guangdong Province, is located at the estuary of the Pearl River in China and is one of the core cities in the Pearl River Delta Gulf region. On February 18, 2019, the State Council officially released the “Guangdong-Hong Kong-Macau Greater Bay Area Development Planning Outline” as a programmatic document to guide the current and future cooperative development of the Guangdong-Hong Kong-Macau Greater Bay Area. The positioning of Guangzhou is as follows: give full play to the leading role of national central cities and comprehensive gateway cities, comprehensively enhance the functions of international business and trade centers and comprehensive transportation hubs, cultivate and improve the functions of science, technology, education, and cultural centers, and strive to build an international metropolis [29]. It can be seen that Guangzhou has a prominent position in the construction and development planning of the Guangdong-Hong Kong-Macau Greater Bay Area; the development of Guangzhou's urban leading industries is crucial to building an international metropolis. Therefore, it is typical and inevitable to choose Guangzhou City (region *i*) and Guangdong Province (the large region where region *i* is located) as research samples.

According to the “*Guangzhou Statistical Yearbook*” data, Guangzhou's GDP has increased from 1,810,041.36 million yuan in 2015 [30] to 2501,910.96 million yuan in 2020 [31], and the total economic volume has grown rapidly. The ratio of the three industrial structures has changed from 0.3 : 29.2 : 70.5 [32] in 2015 to 3.8 : 38.7 : 57.5 [33] in 2020. According to the theory of industrial structure evolution [34], it can be seen that although the tertiary industry first rose and then fell, it has maintained its status as an economic main body, reflecting the transition from the middle stage of industrialization to the later stage of industrialization. Therefore, it is necessary to make partial adjustments to the industrial structure of Guangzhou and to clarify the interrelationship and role of the urban leading industries, pillar industries, and basic industries, so as to ensure a faster, more stable, and healthier growth of Guangzhou's urban economy.

### 3.2. Data Source and Processing

The original data used are from “*Guangzhou Statistical Yearbook* 2016,” “*Guangzhou Statistical Yearbook* 2021,” “*Guangdong Statistical Yearbook* 2016,” and “*Guangdong Statistical Yearbook* 2021.” Based on the consideration of statistical caliber, the caliber of enterprises above the designated size of the total industrial output value of the manufacturing industry is adopted, and the total industrial output value of each industrial sector is the object of analysis. Through the necessary sorting and cleaning of the data of “*China Statistical Yearbook*,” “*Guangdong Statistical Yearbook*,” and “*Guangzhou Statistical Yearbook*,” it is concluded that there are 41 industrial sectors above designated size in the manufacturing industry in Guangdong Province, and, among the 41 industrial sectors in Guangzhou, there are 6 industrial sectors with missing data statistics. Therefore, 35 manufacturing industry sectors in Guangzhou in 2015 and 2020 were selected as the analysis objects, and 35 corresponding industrial sectors in Guangdong Province at the same time points were used as the reference objects. Microsoft Excel 2010 and SPSS 23.0 statistical software were used to process the original data, and SSM mathematical model was used to calculate and analyze the changes and structural characteristics of 35 manufacturing industries in Guangzhou from 2015 to 2020 and get the calculation result.

### 3.3. Analysis of Changes in Industrial Sectors

Calculation was done according to the SSM mathematical model (1)∼(8) to get Tables 1 and 2(*PD*_*ij*_ is the industrial sector advantage index); the analysis of the data in Tables 1 and 2 shows the changes in 35 manufacturing sectors in Guangzhou from 2015 to 2020.

Table 2 shows that there are 26 industrial sectors with *N*_*ij*_ > 0 (accounting for 74%) and 9 industrial sectors with *N*_*ij*_ < 0 (accounting for 26%). It shows that more than 2/3 of the manufacturing industry sectors in Guangzhou have been a growth industry sector in the whole province (Guangdong Province) in recent years, and most of the manufacturing industry sectors have good development prospects; in particular the No. 28 industry sector (*N*_*i*28_ = 236.26) has the fastest growth rate; but, at the same time, nearly 1/3 of the manufacturing industry sectors are showing a recession; in particular the No. 15 industry sector (*N*_*i*15_ = −12.78) has the most obvious recession.

Table 2 shows that there are 25 industrial sectors with *P*_*ij*_ > 0 (accounting for 71%) and 10 industrial sectors with *P*_*ij*_ < 0 (accounting for 29%). It shows that nearly 2/3 of the manufacturing industry sectors in Guangzhou have a good structural foundation, and most of the industrial sectors have the structural foundation advantages in Guangdong Province in recent years; in particular the No. 25 industrial sector (*P*_*i*25_ = 1903.64) has the best structural foundation advantages. But, at the same time, nearly 1/3 of the manufacturing industry sectors have a disadvantageous structural foundation; in particular the No. 15 industrial sector (*P*_*i*15_ = −237.90) has the weakest structural foundation.

Table 2 shows that there are 14 industrial sectors with *D*_*ij*_ > 0 (accounting for 40%) and 21 industrial sectors with *D*_*ij*_ < 0 (accounting for 60%). It shows that 60% of the manufacturing sectors in Guangzhou have weak regional competitiveness in Guangdong Province; in particular the No. 28 industrial sector (*D*_*i*28_ = −1239.28) has the weakest regional competitiveness; only 40% of the industrial sectors have rapid growth and strong regional competitiveness in the province; in particular the No. 19 industrial sector (*D*_*i*19_ = 331.17) has the strongest regional competitiveness.

Table 2 shows that there are 19 industrial sectors with *PD*_*ij*_ > 0 (accounting for 54%) and 16 industrial sectors with *PD*_*ij*_ < 0 (accounting for 46%). It shows tha,t in recent years, Guangzhou's manufacturing industry sector has had a good sectoral advantage in Guangdong Province; in particular the No. 25 industrial sector (*PD*_*i*25_ = 1821.78) has a prominent sectoral advantage; but, at the same time, nearly 1/2 of the manufacturing sectors have a declining advantage; in particular the No. 15 industrial sector (*PD*_*i*15_ = −842.60) has the most severe decline.

### 3.4. Analysis of Characteristics of Overall Industrial Structure

Calculation was done according to the SSM mathematical model (9)∼(17) to get Table 3; Analysis of the data in Table 3 shows the overall industrial structure characteristics of the manufacturing industry in Guangzhou from 2015 to 2020.

Table 3 shows that *G*_*i*_ is larger and *L* < 1, indicating that the economic growth rate of Guangzhou is slower than that of Guangdong Province in the same period, and the overall development speed is relatively slow. In fact, the economic growth of Shenzhen, Dongguan, and Foshan has been particularly rapid in recent years. “*China Economic Weekly*” has published that Shenzhen has surpassed Guangzhou in GDP for five years since the beginning of 2017, ranking first in Guangdong Province.

Table 3 shows that *P*_*i*_ is larger and *W* > 1, indicating that Guangzhou's industrial composition contains a relatively large proportion of sunrise industry sectors, the economic structure is good, and the industrial structure contributes greatly to economic growth.

Table 3 shows that the negative value of *D*_*i*_ is large and *u* < 1, which indicates that many industrial sectors in Guangzhou are developing slowly, showing a declining trend, their status is declining, and their competitiveness is getting weaker and weaker.

## 4. Leading Industry Graphical Analysis and Selection

### 4.1. Shift-Share Analysis

**Figure 1:**
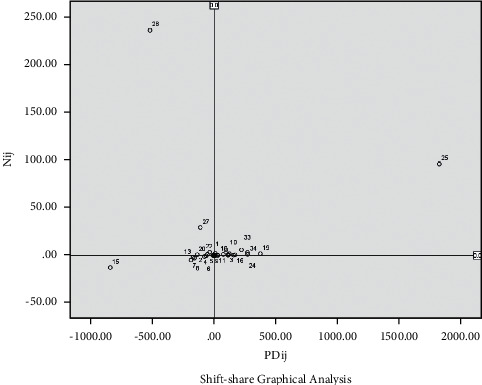
Advantages chart of industrial sectors.

**Figure 2:**
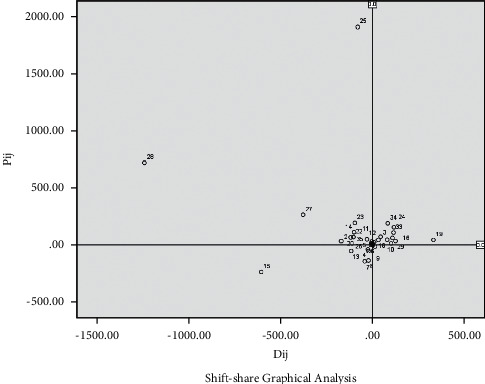
Deviation chart of industrial sectors.

In order to visually display each industrial sector, according to the data in Table 2, SPSS 23.0 statistical software is used to process and generate the industrial sector advantage analysis graph and the industrial sector deviation component graph, as shown in Figures 1 and 2.

According to the analysis theory of Shift-share industrial sector advantage chart [27], the industrial sectors distributed in the first quadrant have both scale growth advantages and industrial sector advantages and belong to good industrial sectors. Although industrial sectors distributed in the second quadrant are growth sectors, they have no sectoral advantages and belong to general industrial sectors. The industrial sectors distributed in the fourth quadrant have sectoral advantages but are declining sectors and also belong to general industrial sectors. The industrial sectors distributed in the third quadrant have neither the advantage of scale growth nor the sectoral advantages, and the industrial sectors belong to the inferior industrial sector. According to Figure 1, there are 19 good industrial sectors, of which the No. 25 industrial sector (*N*_*i*25_ = 96.13, *PD*_*i*25_ = 1821.78) is the best, contributing the most to economic growth, with obvious growth advantages and outstanding sectoral advantages. There are 6 general industrial sectors, of which the No. 28 (*N*_*i*28_ = 236.26, *PD*_*i*28_ = −520.76) industrial sector is typical, although it has good growth advantages but no sectoral advantages. There are 10 poor industrial sectors, of which the No. 15 industrial sector (*N*_*i*15_ = −12.78, *PD*_*i*15_ = −842.60) is the worst, with neither growth advantage nor sectoral advantage.

According to Shift-share industrial sector deviation component diagram analysis theory [27], the industrial sectors distributed in the first quadrant have both structural basic advantages and competitive advantages and belong to excellent industrial sectors; The industrial sectors distributed in the second quadrant have structural foundations advantages but lack competitiveness at the same time; they belong to general industrial sectors. Although industrial sectors distributed in the fourth quadrant have competitive advantages but poor structural foundation, their status is in a downward trend; they also belong to general industrial sectors. The industrial sectors distributed in the third quadrant have neither good structural foundation nor competitiveness and belong to declining industrial sectors. According to Figure 2, there are 12 industrial sectors that belong to rising industrial sectors, of which the No. 12 industrial sector (*P*_*i*12_ = 188.36) has the best structural foundation and No. 6 industrial sector (*D*_*i*6_ = 331.17) is the most competitive. There are 15 sectors belonging to general industries; among them, the No. 25 industrial sector (*P*_*i*25_ = 1903.64) has the best structural foundation and contributes the most to economic growth, and the No. 28 industrial sector (*D*_*i*28_ = −1239.28) has the weakest competitiveness. There are 8 declining industrial sectors, of which the 15 No. industrial sector (*P*_*i*15_ = −237.90, *D*_*i*15_ = −604.71) has the worst structural foundation and lacks competitiveness.

### 4.2. Initial Selection of Leading Industries

According to the SSM algorithm and the three selection criteria, the selection of leading industries should comprehensively consider the industrial sectors with positive and large values of share deviation component, structural deviation component, and competitiveness deviation component. They have fast scale growth, good industrial structure, strong regional competitiveness, outstanding industrial sector advantages, broad development prospects and room for improvement in the present and the next few years, and great potential for technological innovation, which can lead the direction of urban industrial economic development. Therefore, we select 12 common industrial sectors in the first quadrant of Figures 1 and 2 to be preliminarily identified as the city leading industries in Guangzhou, which are 3, manufacture of Food; 10, manufacture of furniture; 12, printing and record medium reproduction; 16, manufacture of medicines; 18, rubber and plastic products; 19, nonmetal mineral products; 21, smelting and pressing of nonferrous metals; 24, manufacture of special-purpose machinery; 29, manufacture of instruments and meters; 32, manufacture of metal products, machinery, and equipment maintenance; 33, production and supply of electric power and heat power; and 34, production and supply of gas.

## 5. Countermeasures and Suggestions for Industrial Development

### 5.1. Accelerate Traditional Industries Technological Upgrading

Among the 12 leading industries identified by the empirical analysis, almost all are traditional industrial sectors, and the traditional industrial sectors will face new pressures and challenges in the future development of new industrialization. Therefore, it is necessary to take precautions to prepare for future challenges in the traditional industry sector. It should adjust, optimize, and upgrade the industrial structure of traditional manufacturing through technological innovation and equipment renewal, vigorously promote and apply advanced and mature electronic information technology, advanced manufacturing technology, bio-engineering technology, energy-saving and consumption-reducing technology, and so on, continuously promote technology research and development and technological progress, inject new vitality into traditional industries, enable them to gain new development, and let the leading industry groups better lead the correct, healthy, and sustainable development of city industries.

### 5.2. Focus on Optimizing Automobile Manufacturing Industry

Through the empirical analysis of Guangzhou's manufacturing industry sector, it is found that the industrial output value of the No. 25 industrial sector “manufacture of automobile” has grown rapidly, and the sector has the strongest advantage among the 35 industrial sectors, whose industrial sector advantage index *PD*_*i*25_ = 1821.78; this is a huge gap with the other 34 industrial sectors. In addition, the structural foundation of the automobile manufacturing industry sector is the best, and the structural deviation component *P*_*i*25_ = 1903.64 is very high, which contributes the most to the economic growth of Guangzhou. According to the data of “*Guangzhou Statistical Yearbook 2016*” and “*Guangzhou Statistical Yearbook 2021*,” the total output value of Guangzhou's automobile manufacturing industry has increased from 393.079 billion yuan in 2015 to 584.870 billion yuan in 2020, accounting for 29% of the total industrial output value and indicating that the automobile manufacturing industry has the greatest contribution and role to Guangzhou's economic development. Unfortunately, the competitiveness of Guangzhou's automobile manufacturing industry is weak (*D*_*i*25_ = −81.86). Therefore, it is necessary to optimize the Guangzhou automobile manufacturing industry by means of talent mechanism, technical mechanism, management mechanism, service mechanism, and resource reintegration mechanism, keeping economic contribution rate and industrial status.

### 5.3. Promote Leading Industries Independent Innovation

The Shift-share analysis chart shows intuitively that nearly half of the 35 manufacturing sectors belong to the midstream sector, and nearly 1/3 of the sectors belong to recession sectors. Insufficient innovation capability in most industrial sectors leads to lack of sectoral advantages and lack of competitiveness, which is necessary to adjust the industrial structure of the middle and downstream industrial sectors, and at the same time gives full play to the radiation and leading role of leading industries. Therefore, it is necessary to further establish the important strategic position of high-tech industries in Guangzhou's economic development, increase investment in technology research and development, promote independent technological innovation in leading industrial enterprises, establish and improve technology research and development institutions, and promote technological development and innovation. Only by allowing leading industries to take the lead in possessing high-level advanced science and technology and by industrializing advanced scientific and technological achievements, through interindustry diffusion and radiation, can the development of mid-downstream industrial sectors be driven and the quality and level of mid-downstream industrial sectors be improved, and only with the guiding role of structural optimization can the midstream and downstream industrial sectors gain new opportunities for development.

### 5.4. Create Leading Industry Sharing Platform

Building a development platform for resource sharing is the key to attracting talents and customers; the healthy development direction of the city's leading industries in the future should be a scientific and circular development model that seeks the harmonious coexistence of people, resources, and the environment at the center, achieve the greatest economic effect with the least resources, and realize the human, resources, and environment with a shared platform. So creating a good platform environment for the development of leading industries can bring social resources to Guangzhou and accelerate the development of leading industries in the city. Therefore, various platforms need to be built and cultivated for the development of leading industries by integrating social resources in Guangzhou, including financial investment platforms, information exchange platforms, technology research and development platforms, human resource development platforms, and market development platforms, and a sound industrial service system needs to be established, as well as an incentive mechanism, to provide a full range of services for the development of leading industries. At the same time, it is necessary to strengthen industrial cooperation, infrastructure cooperation, and market sharing between cities, make full use of the advanced manufacturing market space and high-tech achievements in the Guangdong-Hong Kong-Macao Greater Bay Area to develop leading industries, and increase the construction of transportation and information networks, as well as to eliminate local protectionism and administrative barriers to promote resource sharing, market access, coordinated regional economic development, and win-win cooperation.

## 6. Conclusion

The deviation-share analysis method based on the SSM algorithm is a comprehensive method with strong objectivity, dynamics, and quantification; it is also an effective method to reveal the reasons for the structural changes of cities and urban industrial sectors and then to determine the leading direction of the future development of city industries. Although this method is relatively mature and complete in quantitative analysis and reflects good dynamics and objectivity, it is still the embodiment of the idea of “material-oriented economy” with efficiency as the center and the growth of social material wealth as the ultimate goal (social environment and natural environment) being not concerned. It ignores the systematic consideration of the relationship between the industrial economy and people's urban life and the environment and also ignores the “human-oriented economy” thought of people's participation in social and economic growth and the comfort of environmental experience. Selecting and determining the leading industry will lead to problems such as unbalanced development of regional industrial economy, uncoordinated economic development, and environmental development. Therefore the SSM mathematical model and the Shift-share analysis chart are used as empirical analysis methods to filter out the leading industrial clusters in Guangzhou; the 12 leading industrial sectors preliminarily determined do not absolutely represent the final leading industries in Guangzhou. However, the final determination of the leading industry also needs to consider other indicators such as environmental harmony, industrial association, and technical level, especially the level of high-tech-cutting-edge technologies. Therefore, it is necessary to increase the construction of high-tech-cutting-edge technology industrial parks, establish a new mechanism for scientific and technological talents, speed up the introduction of high-tech talents and the development of high-tech-cutting-edge technology, and accelerate the pace of industrialization of high-tech-cutting-edge technology in order to better lead the urban industrial economy to develop steadily forward with the core role of the leading industry.

## Figures and Tables

**Table 1:** The variation (rate) in all manufacturing industries of Guangzhou in 2015～2020.

Number	*b*_*ij*, 0_	*b*_*ij*, *t*_	*B*_*j*, 0_	*B*_*j*, *t*_	*r*_*ij*_	*R*_*j*_	*b´*_*ij*_
1	2.24	0.46	385.54	213.76	−0.79	−0.45	0.01
2	465.67	328.83	3130.38	3356.27	−0.29	0.07	11.77
3	474.35	589.19	1805.54	2079.62	0.24	0.15	6.91
4	300.93	234.92	1146.1	993.06	−0.22	−0.13	2.78
5	217.63	222.65	460.9	487.82	0.02	0.06	0.81
6	266.91	187.32	2639.51	2077.61	−0.30	−0.21	5.69
7	422.79	229.52	4073.91	2633.34	−0.46	−0.35	13.90
8	289.31	126.64	2460.87	1271.79	−0.56	−0.48	5.75
9	32.09	29.29	841.51	444.2	−0.09	−0.47	0.22
10	177.33	289.96	1873.33	2003.68	0.64	0.07	2.68
11	132.11	158.5	2013.29	2483.28	0.20	0.23	2.15
12	99.38	108.43	1218.89	1319.52	0.09	0.08	0.98
13	320.8	147.47	3934.3	3244.91	−0.54	−0.18	10.19
14	504.07	515.18	2331.04	2853.29	0.02	0.22	9.48
15	2069.81	1214.43	6315.93	5551.01	−0.41	−0.12	105.51
16	244.73	414.37	1484.49	1859.72	0.69	0.25	2.93
17	6.61	5.88	128.15	178.97	−0.11	0.40	0.01
18	388.5	513.5	4860.96	5440.54	0.32	0.12	15.24
19	178.61	554.71	5007.36	6266.94	2.11	0.25	7.22
20	361.15	308.07	2332.54	2765.22	−0.15	0.19	6.80
21	415.95	491.33	3181.13	3519.29	0.18	0.11	10.68
22	376.3	342.89	5855.38	6974.39	−0.09	0.19	17.78
23	653.66	754.24	3646.22	4750.4	0.15	0.30	19.24
24	200.4	473.33	2479.64	4425.33	1.36	0.78	4.01
25	3930.79	5848.7	5955.96	8986.03	0.49	0.51	188.96
26	435.47	425.27	1241.12	1240.53	−0.02	0.00	4.36
27	1080.89	997.33	12428.41	15803.07	−0.08	0.27	108.43
28	2407.42	2122.92	30658.71	42817.87	−0.12	0.40	595.72
29	64.97	223.03	866.87	1313.68	2.43	0.52	0.45
30	18.54	24.55	253.44	397.1	0.32	0.57	0.04
31	28.39	24.38	1151.96	980.08	−0.14	−0.15	0.26
32	56.37	86.18	133.86	189.4	0.53	0.41	0.06
33	1395.47	1622.53	6405.36	6925.33	0.16	0.08	72.14
34	317.22	588.87	789.54	1261.35	0.86	0.60	2.02
35	87.88	105.28	405.29	630.42	0.20	0.56	0.29

The number corresponds to the industry sector: 1, mining and dressing of nonmetal ores; 2, processing of farm and sideline food; 3, manufacture of food; 4, manufacture of wine, beverage, and refined tea; 5, tobacco products; 6, textile industry; 7, manufacture of textile garments, footwear, and headgear; 8, Leather, fur, feather, down, and related products; 9, timber processing, bamboo, cane, palm fiber, and straw products; 10, manufacture of furniture; 11, papermaking and paper products; 12, printing and record medium reproduction; 13, manufacture of cultural, educational, sports, and entertainment articles; 14, petroleum refining, coking, and nuclear fuel processing; 15, manufacture of raw chemical materials and chemical products; 16, manufacture of medicines; 17, manufacture of chemical fibers; 18, rubber and plastic products; 19, nonmetal mineral products; 20, smelting and pressing of ferrous metals; 21, smelting and pressing of nonferrous metals; 22, metal products; 23, manufacture of general-purpose machinery; 24, manufacture of special-purpose machinery; 25, manufacture of automobile; 26, manufacture of railway, ship, aeronautics, and other transport equipment; 27, manufacture of electrical machinery and equipment; 28, manufacture of communication equipment, computers, and other electronic equipment; 29, manufacture of instruments and meters; 30, other manufactures (crafts); 31, comprehensive utilization of waste; 32, manufacture of metal products, machinery, and equipment maintenance; 33, production and supply of electric power and heat power; 34, production and supply of gas; and 35, production and supply of water.

**Table 2:** The changes in all manufacturing industries of Guangzhou in 2015～2020.

Number	*G*_*ij*_	*N*_*ij*_	*P*_*ij*_	*D*_*ij*_	*PD*_*ij*_
1	−1.78	−0.0031	−0.99	−0.78	−1.78
2	−136.84	0.85	32.75	−170.44	−137.69
3	114.84	1.05	70.96	42.83	113.79
4	−66.01	−0.37	−39.81	−25.83	−65.64
5	5.02	0.05	12.66	−7.69	4.97
6	−79.59	−1.21	−55.61	−22.77	−78.38
7	−193.27	−4.92	−144.59	−43.77	−188.35
8	−162.67	−2.78	−137.02	−22.88	−159.89
9	−2.80	−0.10	−15.05	12.35	−2.70
10	112.63	0.19	12.15	100.29	112.44
11	26.39	0.50	30.34	−4.45	25.89
12	9.05	0.08	8.12	0.85	8.97
13	−173.33	−1.78	−54.43	−117.12	−171.55
14	11.11	2.12	110.81	−101.82	8.99
15	−855.38	−12.78	−237.90	−604.71	−842.60
16	169.64	0.74	61.12	107.78	168.90
17	−0.73	0.0027	2.62	−3.35	−0.73
18	125.00	1.82	44.50	78.68	123.18
19	376.10	1.82	43.11	331.17	374.28
20	−53.08	1.26	65.73	−120.07	−54.34
21	75.38	1.14	43.08	31.16	74.24
22	−33.41	3.40	68.52	−105.32	−36.81
23	100.58	5.83	192.12	−97.37	94.75
24	272.93	3.15	154.10	115.68	269.78
25	1917.91	96.13	1903.64	−81.86	1821.78
26	−10.20	−0.0021	−0.20	−9.99	−10.20
27	−83.56	29.44	264.05	−377.05	−113.00
28	−284.50	236.26	718.51	−1239.28	−520.76
29	158.06	0.23	33.25	124.57	157.83
30	6.01	0.02	10.49	−4.50	5.99
31	−4.01	−0.04	−4.20	0.23	−3.97
32	29.81	0.03	23.36	6.42	29.78
33	227.06	5.86	107.42	113.78	221.20
34	271.65	1.21	188.36	82.09	270.44
35	17.40	0.16	48.66	−31.42	17.24

The number corresponds to the industry sector: 1, mining and dressing of nonmetal ores; 2, processing of farm and sideline food; 3, manufacture of food; 4, manufacture of wine, beverage, and refined tea; 5, tobacco products; 6, textile industry; 7, manufacture of textile garments, footwear, and headgear; 8, Leather, fur, feather, down, and related products; 9, timber processing, bamboo, cane, palm fiber, and straw products; 10, manufacture of furniture; 11, papermaking and paper products; 12, printing and record medium reproduction; 13, manufacture of cultural, educational, sports, and entertainment articles; 14, petroleum refining, coking, and nuclear fuel processing; 15, manufacture of raw chemical materials and chemical products; 16, manufacture of medicines; 17, manufacture of chemical fibers; 18, rubber and plastic products; 19, nonmetal mineral products; 20, smelting and pressing of ferrous metals; 21, smelting and pressing of nonferrous metals; 22, metal products; 23, manufacture of general-purpose machinery; 24, manufacture of special-purpose machinery; 25, manufacture of automobile; 26, manufacture of railway, ship, aeronautics, and other transport equipment; 27, manufacture of electrical machinery and equipment; 28, manufacture of communication equipment, computers, and other electronic equipment; 29, manufacture of instruments and meters; 30, other manufactures (crafts); 31, comprehensive utilization of waste; 32, manufacture of metal products, machinery, and equipment maintenance; 33, production and supply of electric power and heat power; 34, production and supply of gas; and 35, production and supply of water.

**Table 3:** The total effective index in all manufacturing industries of Guangzhou in 2015～2020.

*G*_*i*_	*N*_*i*_	*P*_*i*_	*D*_*i*_	*L*	*W*	*u*
1885.41	369.34	3560.65	−2044.58	0.92	1.02	0.91

## Data Availability

The dataset can be accessed upon request.
